# Finite Element Approach for the Simulation of Modern MRAM Devices

**DOI:** 10.3390/mi14050898

**Published:** 2023-04-22

**Authors:** Simone Fiorentini, Nils Petter Jørstad, Johannes Ender, Roberto Lacerda de Orio, Siegfried Selberherr, Mario Bendra, Wolfgang Goes, Viktor Sverdlov

**Affiliations:** 1 Christian Doppler Laboratory for Nonvolatile Magnetoresistive Memory and Logic at the Institute for Microelectronics, TU Wien, Gußhausstraße 27-29/E360, 1040 Vienna, Austria; fiorentini@iue.tuwien.ac.at (S.F.); jorstad@iue.tuwien.ac.at (N.P.J.); ender@iue.tuwien.ac.at (J.E.); bendra@iue.tuwien.ac.at (M.B.); 2 Institute for Microelectronics, TU Wien, Gußhausstraße 27-29/E360, 1040 Vienna, Austria; orio@iue.tuwien.ac.at (R.L.d.O.); selberherr@tuwien.ac.at (S.S.); 3 Silvaco Europe Ltd., Cambridge PE27 5JL, UK; wolfgang.goes@silvaco.com

**Keywords:** finite element method, micromagnetics, spin and charge drift-diffusion, MRAM

## Abstract

Because of their nonvolatile nature and simple structure, the interest in MRAM devices has been steadily growing in recent years. Reliable simulation tools, capable of handling complex geometries composed of multiple materials, provide valuable help in improving the design of MRAM cells. In this work, we describe a solver based on the finite element implementation of the Landau–Lifshitz–Gilbert equation coupled to the spin and charge drift-diffusion formalism. The torque acting in all layers from different contributions is computed from a unified expression. In consequence of the versatility of the finite element implementation, the solver is applied to switching simulations of recently proposed structures based on spin-transfer torque, with a double reference layer or an elongated and composite free layer, and of a structure combining spin-transfer and spin-orbit torques.

## 1. Introduction

As the scaling of conventional CMOS technology shows signs of saturation, due to the increase in standby power consumption and leakage currents, the employment of nonvolatile memory components which do not require the memory bits to be refreshed becomes increasingly appealing [1]. One of the most promising candidates as a nonvolatile replacement is magnetoresistive random access memory (MRAM). It possesses a simple structure and is directly compatible with CMOS back-end of line processes. It has shown to be promising for several applications, for example in stand-alone memories and in the embedded automotive and Internet of Things fields, and is expected to replace charge-based devices in frame buffer memory and slow SRAM [2,3,4,5]. Moreover, MRAM devices have shown to be interesting for cryogenic applications, especially for employment in quantum computing systems [6,7,8].

The core of an MRAM cell is the magnetic tunnel junction (MTJ), a stack of two ferromagnetic (FM) layers separated by an oxide layer. The properties of the two FM layers are such that the magnetization in one of them, the reference layer (RL), is fixed, while in the other one, the free layer (FL), it can be switched between the two stable parallel (P) and anti-parallel (AP) states. The resistance of the stack can be employed to store the binary information, as it is higher in the AP state. The percentage difference between the resistance of the two stable states is labeled the tunneling magnetoresistance ratio (TMR).

**Figure 1:**
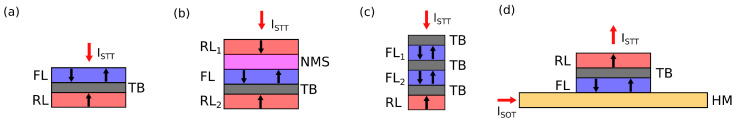
Four examples of multi-layer MRAM cell design: (**a**) standard STT-MRAM with single MTJ; (**b**) double RL STT-MRAM, where the second RL provides additional torque to reduce the critical voltage required for switching [20]; (**c**) ultra-scaled STT-MRAM, where the FM layers are elongated and additional oxide layers are added to improve scalability and benefit from the shape anisotropy [21]; (**d**) SOT-assisted STT-MRAM, where the switching process is kick-started by an initial current pulse in the HM [22].

The writing process in modern devices is performed by relying on spin-transfer torque (STT), spin-orbit torque (SOT), or a combination of both of them. In STT switching, the torque is generated by a current flowing through the MTJ. The electrons are polarized by the RL and transfer their polarization to the FL magnetization, providing the torque [9,10,11]. Examples of structures based on STT are reported in Figure 1a–c. In SOT switching, the torque is generated by passing the current through a heavy metal line (HM) below the FL. The spin Hall effect (SHE) produces a spin current orthogonal to the charge one, which is absorbed by the FL magnetization, providing the torque [12,13]. An example of a three-terminal structure combining STT and SOT is shown in Figure 1d.

The design of modern MRAM cells can be supported by the development of reliable simulation tools. The magnetization dynamics are described by the Landau–Lifshitz–Gilbert (LLG) equation, which must be supplied with a term describing the spin torque. The drift-diffusion formalism offers a way of computing different torque contributions in all the ferromagnetic layers from a unified expression [14,15,16]. The finite element (FE) method, being able to handle structures with several domains of different materials and complex geometries, represents an optimal choice for computing a numerical solution to the micromagnetic equations in modern MRAM cells. In this work, we present an FE-based implementation of the LLG equation coupled with the drift-diffusion formalism, extended to include the charge and torque properties expected in MTJs. The solver was developed by employing the open-source C++ FE library MFEM [17,18], and is applied to the simulation of recently proposed structures based both on STT and SOT switching. The source code is available as an open-source repository [19].

## 2. Micromagnetic Modeling

The LLG equation for the description of the magnetization dynamics was first derived by Landau and Lifshitz in 1935 [23] and reworked by Gilbert in a more treatable form in 1955 [24]. With the inclusion of the spin torque $T_{S}$, it takes the form

(1) $$\frac{\partial m}{\partial t}=-\left|\gamma \right|\mu_{0}\;m\times H_{\mathrm{eff}}+\alpha \;m\times \frac{\partial m}{\partial t}+\frac{1}{M_{S}}T_{S}$$

where $m=M/M_{S}$ is the unit vector in the direction of the local magnetization, $M_{S}$ is the saturation magnetization, γ is the gyromagnetic ratio, and $\mu_{0}$ is the vacuum permeability. $H_{\mathrm{eff}}$ is an effective magnetic field including the contributions of an externally applied field, the exchange coupling, the anisotropy field, and the demagnetizing field. The effects of temperature, which can be included by an additional effective field contribution describing thermal fluctuations [25], are not considered for the switching results presented in this work. The main effect of their inclusion would be to reduce the incubation time necessary for the switching process, while the behavior would remain qualitatively similar. While the external field $H_{\mathrm{ext}}$ can be simply added as an input parameter, the other contributions are intrinsic to the ferromagnets and must be computed from material parameters.

The exchange coupling can be modeled through a field which tends to keep the magnetization vectors aligned throughout the magnetic domain, described by the expression

(2) $$H_{\mathrm{exc}}=\frac{2A_{\mathrm{exc}}}{\mu_{0}M_{S}}\nabla^{2}m\;,$$

where $A_{\mathrm{exc}}$ is the exchange coefficient.

Modern MRAM cells utilize MTJs with magnetization perpendicular to the stack, by virtue of both interface and shape anisotropy contributions [21]. While the latter is taken into account by the demagnetizing field, the former can be included as a uniaxial anisotropy field with the expression

(3) $$H_{\mathrm{ani}}=\frac{2K_{\mathrm{ani}}}{\mu_{0}M_{S}}\left(a\cdot m\right)a\;,$$

where a is a unit vector in the direction perpendicular to the stack and $K_{\mathrm{ani}}$ is the anisotropy coefficient, which can be computed from the interface anisotropy $K_{\mathrm{int}}$ as $K_{\mathrm{ani}}=K_{\mathrm{int}}/d_{\mathrm{FM}}$, where $d_{\mathrm{FM}}$ is the thickness of the ferromagnetic layer under consideration.

The demagnetizing field can be computed from the scalar magnetic potential $u_{m}$ as

(4) $$H_{\mathrm{demag}}=-\nabla u_{m}$$

$u_{m}$ is obtained through the solution of the Poisson equation

(5) $$-\nabla^{2}u_{m}=-M_{S}\nabla \cdot m\;,$$

with the constraint of $u_{m}$ decaying to zero as $O(1/|x{|}^{2})$ outside the magnetic domain and the boundary condition $[\nabla u_{m}\cdot n]=-M_{S}\;m\cdot n$, where n is the unit vector normal to the boundary and […] denotes a discontinuity across the boundary.

In the presence of a single thin FL, the torque acting on the magnetization can be described by simplified expressions [26,27,28,29], derived by Slonczewski [9,11]. A more general form of the torque term, which allows an arbitrary number of ferromagnetic and non-magnetic layers to be dealt with, can be obtained by computing the non-equilibrium spin accumulation in the structure under study through the solution of spin and charge transport equations.

### Spin and Charge Transport

The torque term $T_{S}$ entering (1) can be computed from the spin accumulation through the following expression [16,30,31,32]:

(6) $$T_{S}=-D_{e}\frac{m\times S}{\lambda_{J}^{2}}-D_{e}\frac{m\times \left(m\times S\right)}{\lambda_{\varphi }^{2}}$$

The first term describes the precession around the exchange field and is characterized by the exchange length $\lambda_{J}$, and the second term describes the dephasing process of the spins of the transiting electrons, and is characterized by the dephasing length $\lambda_{\varphi }$. $D_{e}$ is the electron diffusion coefficient. The spin accumulation S describes the deviation of the polarization of the conducting electrons from the equilibrium configuration created by a charge current density $J_{C}$, in units of the transported magnetic moment (A/m). Thus, by definition, S is non-zero only when an electric current is flowing through the system [33]. A solution for S in all non-magnetic and ferromagnetic layers of an MRAM cell can be obtained by means of the spin and charge drift-diffusion formalism.

Spin and charge drift-diffusion equations in multilayer structures with arbitrary magnetization orientation were reported by S. Zhang, P. Levy, and A. Fert [34], with both the precession and decay of the transverse spin accumulation components governed by the exchange length $\lambda_{J}$. Another possible mechanism for the absorption of the transverse components is the dephasing process [32,35]. The behavior of the spin accumulation with both precessional and dephasing terms was described in terms of the Continuous Random Matrix Theory (CRMT) in [35], and the equivalence of the CMRT and the spin and charge drift-diffusion formalism was shown. The resulting equations for spin and charge currents are [14,16]:

(7) $$\begin{array}{cc} J_{C} & =\sigma E+\frac{e}{\mu_{B}}\beta_{D}D_{e}{\left(\nabla S\right)}^{T}m\;, \end{array}$$

(8) $$\begin{array}{cc} {\tilde{J}}_{S} & =-\frac{\mu_{B}}{e}\beta_{\sigma }m⊗\left(\sigma E\right)-D_{e}\nabla S\;, \end{array}$$

where ${\tilde{J}}_{S}$ is the spin current tensor, $J_{C}$ is the charge current density, $\mu_{B}$ is the Bohr magneton, *e* is the elementary charge, σ is the conductivity, and ⊗ is the outer product. $\beta_{\sigma }=\left(\sigma^{↑}-\sigma^{↓}\right)/\left(\sigma^{↑}+\sigma^{↓}\right)$ and $\beta_{D}=\left(D_{e}^{↑}-D_{e}^{↓}\right)/\left(D_{e}^{↑}+D_{e}^{↓}\right)$ are the conductivity and diffusion polarization parameters, respectively, with $\sigma^{↑}$, $D_{e}^{↑}$ ($\sigma^{↓}$, $D_{e}^{↓}$) the conductivity and diffusion coefficient for the majority (minority) electrons. ∇S is the vector gradient of S, with components ${\left(\nabla S\right)}_{\mathrm{ij}}=\partial S_{i}/\partial x_{j}$, and the term ${\left(\nabla S\right)}^{T}m$ is a vector with components ${\left({\left(\nabla S\right)}^{T}m\right)}_{i}=\sum_{j}\left(\partial S_{i}/\partial x_{j}\right)m_{j}$. The spin current can be expressed in terms of the charge current by inserting (7) into (8):

(9) $${\tilde{J}}_{S}=-\frac{\mu_{B}}{e}\beta_{\sigma }m⊗\left(J_{C}-\frac{e}{\mu_{B}}\beta_{D}D_{e}{\left(\nabla S\right)}^{T}m\right)-D_{e}\nabla S$$

The equation of motion for the spin accumulation is given by

(10) $$\frac{\partial S}{\partial t}=-\nabla \cdot {\tilde{J}}_{S}-D_{e}\frac{S}{\lambda_{sf}^{2}}-T_{S}\;,$$

where $\lambda_{\mathrm{sf}}$ is the spin-flip length and $\nabla \cdot {\tilde{J}}_{S}$ is the divergence of ${\tilde{J}}_{S}$, with components ${\left(\nabla \cdot {\tilde{J}}_{S}\right)}_{i}=\sum_{j}\partial J_{S,\mathrm{ij}}/\partial x_{j}$. As the typical time scale for the magnetization motion is three orders of magnitude larger than the spin accumulation one [34], it is sufficient to consider a steady-state expression for the spin accumulation. This assumption was numerically verified in [36]. With ∂S/∂t=0, the equation describing the spin accumulation becomes

(11) $$-\nabla \cdot {\tilde{J}}_{S}-D_{e}\frac{S}{\lambda_{sf}^{2}}-T_{S}=0$$

## 3. Finite Element Implementation

The presented set of equations allows the magnetization dynamics of structures containing an arbitrary number of layers of different materials to be described. The FE method, a numerical approach for the computation of approximate solutions to partial differential equations, is naturally able to handle meshes with complex geometries and several domains of different materials [37,38], and was therefore employed for the implementation of a solver capable of handling charge, spin accumulation, and the magnetization dynamics. The implementation was carried out by employing the open-source C++ FE library MFEM [17,18].

The first schemes for a FE implementation of the LLG equation, which considered only the contribution of the exchange coupling, were proposed in [39,40]. A new FE algorithm, referred to as the tangent plane integrator scheme, was introduced in [41] and later generalized in [42,43] to include the contributions of the demagnetizing and anisotropy fields. The unconditional convergence of an algorithm coupling the LLG equation with a FE implementation of the spin and charge drift-diffusion formalism was proven in [44], and the scheme was later successfully applied to metallic spin valves in [14]. We report here an extension of the scheme to MTJs, which includes the spin dephasing contribution and allows both the TMR effect and the expected torque properties to be reproduced [45,46].

### 3.1. Charge Current Solution

For the computation of the charge current entering (9), the Laplace equation is solved:

(12) $$\begin{array}{c} -\nabla \cdot \left(\sigma \nabla V\right)=0 \end{array}$$

(13) $$\begin{array}{c} J_{C}=-\sigma \nabla V \end{array}$$

where *V* is the electric potential. Dirichlet conditions are applied to prescribe the voltage at the contacts, and the Neumann condition σ∇V·n=0 is assumed on external boundaries not containing an electrode, with n the unit vector normal to the boundary. In order to be able to reproduce the TMR effect, the tunnel barrier is treated as a poor conductor whose local conductivity depends on the relative magnetization orientation in the RL and FL [45,46]:

(14) $$\sigma_{\mathrm{TB}}=\sigma_{0}\left(1+P_{\mathrm{FL}}\;P_{\mathrm{RL}}m_{\mathrm{RL}}\cdot m_{\mathrm{FL}}\right)$$

where $P_{\mathrm{RL}}$ and $P_{\mathrm{FL}}$ are the Slonczewski polarization parameters [11,47], $\sigma_{0}=\left(\sigma_{P}+\sigma_{AP}\right)/2$ is the angle independent portion of the conductivity, $\sigma_{P(AP)}$ is the conductivity in the parallel (anti-parallel) state, and $m_{\mathrm{RL}(\mathrm{FL})}$ is the magnetization of the RL(FL) close to the interface. $P_{\mathrm{RL}}$ and $P_{\mathrm{FL}}$ are related to the TMR by Julliere’s formula [48]:

(15) $$\mathrm{TMR}=\frac{R_{\mathrm{AP}}-R_{P}}{R_{P}}=\frac{2\;P_{\mathrm{FL}}P_{\mathrm{RL}}}{1-P_{\mathrm{FL}}P_{\mathrm{RL}}}$$

where $R_{P(\mathrm{AP})}$ is the resistance in the parallel (anti-parallel) state.

In order to derive an FE representation, the equations must be written in the so-called weak formulation. For the presented Laplace equation, by using Gauss’s theorem and applying the Neumann boundary conditions, the weak form reduces to

(16) $$\int_{\Omega }\sigma \nabla V\cdot \nabla vdx=0$$

The test function *v* and the solution are both assumed to belong to the Sobolev space $H^{1}$, so that both they and their weak gradients are $L^{2}$-integrable [43].

In the FE approximation, in order to obtain a discretized version of (16), the original domain Ω is divided into smaller regular elements. The discrete solution $V_{h}$ is defined by its values on the elements’ nodes:

(17) $$V_{h}\left(x\right)=\sum_{i=1}^{N}V_{i}\varphi_{i}\left(x\right)$$

where *N* is the total number of nodes, $V_{i}=V_{h}\left(x_{i}\right)$ are the values assumed by the approximate solution at the nodes, and $x_{i}$ is the coordinate vector of node i. $\varphi_{i}$ is an affine function of the nodal basis of the mesh, characterized as

(18) $$\varphi_{i}\left(x_{j}\right)=\delta_{ij}$$

**Figure 2:**
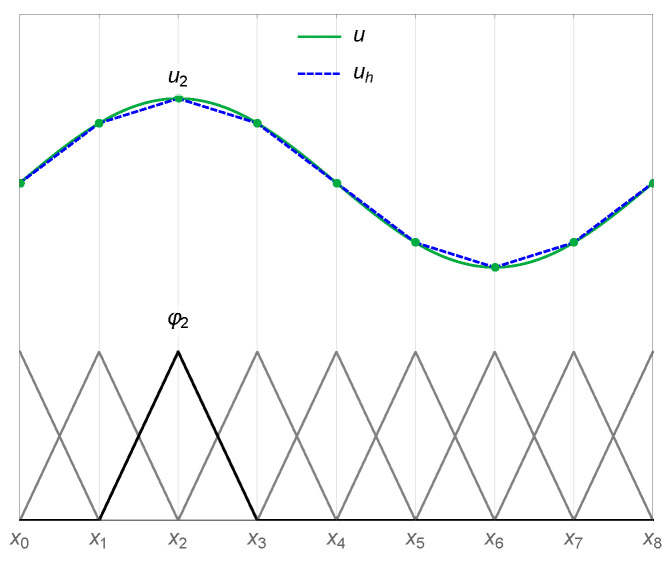
Representation of a continuous function *u* and its finite element approximation $u_{h}$ in a one-dimensional setting. The basis functions for all the nodes are reported at the bottom of the graph. The basis function and solution value associated with the node $x_{2}$ are labeled $\varphi_{2}$ and $u_{2}$, respectively.

Figure 2 illustrates an example of the approximation of a function *u* through linear basis functions in a one-dimensional scenario.

With the given nodal basis decomposition, the original problem can thus be rewritten as the following system of linear equations:

(19) $$\underline{A_{V}}\;\underline{V_{h}}=\underline{0}$$

where $\underline{A_{V}}\in R^{N}\times R^{N}$ is the matrix representation of the left-hand side (LHS) of (16), and $\underline{V_{h}}$ is a vector in $R^{N}$ composed of the nodal values of $V_{h}$. As only neighboring nodes have overlapping basis functions, $\underline{A_{V}}$ is a sparse matrix, with non-zero terms only around the diagonal.

The weak formulation of (13) is

(20) $$\int_{\Omega }J_{C}\cdot vdx=-\int_{\Omega }\sigma \nabla V\cdot vdx$$

By choosing the test function v so that its components belong to $H^{1}$, noting the space containing v as $H^{1}$, this equation allows a projection to be obtained of $J_{C}$ in the $H^{1}$ function space [33] so that it can be readily employed for the computation of the spin accumulation. The resulting system of linear equations is

(21) $$\underline{A_{J}}\;\underline{J_{C,h}}=\underline{f_{J}}\;,$$

where $\underline{A_{J}}\in R^{3N}\times R^{3N}$ is the matrix coming from the LHS of (20) and $\underline{f_{J}}\in R^{3N}$ is the vector coming from the right-hand side (RHS).

The solution to both systems of equations is computed through a solver based on the conjugate gradient method [49], designed for the numerical solution of systems of linear equations whose matrices are positive definite, provided by the library MFEM.

In the scope of the MFEM library, only the data associated with a local element can be accessed during the assembly of the system matrices, while the computation of (14) in the TB requires knowledge of the magnetization vectors in the neighboring FM layers. In order to obtain access to the magnetization values, the coefficient describing the TB conductivity is initialized as follows:

- For each point inside the TB where the conductivity needs to be computed, referred to as an integration point, the solver loops through the integration points of the RL and FL elements closer to the interfaces.
- The RL and FL points near to or at the interface with coordinates closest to the TB point are selected.
- The integration point number and element number associated with the nearest RL and FL points are mapped to the coordinates of the TB points.

In a transient simulation, the search is carried out only during the initialization of the solver. At every time step, the data necessary for the computation of (14) can be accessed through the generated maps, without the need to repeat the search procedure. The computed charge current, consistent with the TMR effect, can then be employed to obtain a solution to the spin accumulation equation.

### 3.2. Spin Accumulation Solution

The weak form of Equation (11), with the spin current expressed as (9) and the spin torque as (6), takes the form

(22) $$\begin{array}{c} -D_{e}\int_{\Omega }\left(\nabla \cdot \left(\nabla S-\beta_{\sigma }\beta_{D}m⊗\left({\left(\nabla S\right)}^{T}m\right)\right)\right)\cdot vdx+\\ +D_{e}\int_{\Omega }\left(\frac{S}{\lambda_{sf}^{2}}+\frac{S\times m}{\lambda_{J}^{2}}+\frac{m\times \left(S\times m\right)}{\lambda_{\varphi }^{2}}\right)\cdot vdx=\\ \frac{\mu_{B}}{e}\beta_{\sigma }\int_{\omega }\left(\nabla \cdot \left(m⊗J_{C}\right)\right)\cdot vdx\;, \end{array}$$

where v represents again a test function belonging to $H^{1}$, S also belongs to $H^{1}$, and ω indicates a subdomain composed of only the ferromagnetic layers. By applying Gauss’s theorem, the first term on the LHS of (22) becomes

(23) $$\begin{array}{c} D_{e}\int_{\Omega }\left(\nabla S-\beta_{\sigma }\beta_{D}m⊗\left({\left(\nabla S\right)}^{T}m\right)\right):\nabla vdx+\\ -\int_{\partial \Omega }\left(\left(\nabla S\right)n-\beta_{\sigma }\beta_{D}\left(m⊗m\right)\left(\left(\nabla S\right)n\right)\right)\cdot vdx\;, \end{array}$$

where $\nabla a:\nabla b=\sum_{\mathrm{ij}}\left(\partial a_{i}/\partial x_{j}\right)\left(\partial b_{i}/\partial x_{j}\right)$ is the Frobenius inner product of two matrices. By assuming the natural Neumann condition $\left(\nabla S\right)n=0$ on all external boundaries of the whole domain Ω, the integrals on ∂Ω are put to zero. If the contacts are longer than the spin-flip length, the condition is equivalent to an exponential decay of S towards the electrodes [14,16].

Gauss’s theorem can also be applied to the RHS term of (22), obtaining

(24) $$-\frac{\mu_{B}}{e}\beta_{\sigma }\int_{\omega }\left(m⊗J_{C}\right):\nabla vdx+\frac{\mu_{B}}{e}\beta_{\sigma }\int_{\partial \Omega \cap \partial \omega }\left(\left(m⊗J_{C}\right)n\right)\cdot vdx$$

∂ω indicates the external boundaries of the magnetic subdomains, and ∂Ω∩∂ω indicates the shared external boundaries of the subdomain ω and the whole domain Ω.

#### 3.2.1. Tunneling Spin Current

The inclusion of appropriate boundary conditions at the TB interface with the RL and FL, together with the employment of a low diffusion coefficient inside the TB, allows the expected properties of the torque acting in MTJs to be reproduced [46]. The additional boundary conditions to be added to the RHS of (22) read

(25) $${\mathrm{RHS}}_{\mathrm{TB}}=-\int_{RL|TB}J_{S,\mathrm{TB}}\cdot v\;dx+\int_{TB|FL}J_{S,\mathrm{TB}}\cdot v\;dx\;,$$

where RL|TB(TB|FL) indicates the interface of the TB with the RL(FL). These internal boundary conditions prescribe the difference in spin current between the FM layers and the TB, according to the spin current polarization generated by the tunneling process. The tunneling spin current $J_{S,\mathrm{TB}}$ can be expressed as [47,50]

(26) $$\begin{array}{c} J_{S,\mathrm{TB}}=-\frac{\mu_{B}}{e}\;\frac{J_{C,\mathrm{TB}}\cdot n}{1+P_{\mathrm{RL}}\;P_{\mathrm{FL}}\;m_{\mathrm{RL}}\cdot m_{\mathrm{FL}}}(a_{\mathrm{mx}}\;P_{\mathrm{RL}}\;m_{\mathrm{RL}}+a_{\mathrm{mx}}\;P_{\mathrm{FL}}\;m_{\mathrm{FL}}+\\ \left.+1/2\;\left(P_{\mathrm{RL}}\;P_{\mathrm{RL}}^{\eta }-P_{\mathrm{FL}}\;P_{\mathrm{FL}}^{\eta }\right)\;m_{\mathrm{RL}}\times m_{\mathrm{FL}}\right)\;, \end{array}$$

where $P_{RL}^{\eta }$ and $P_{FL}^{\eta }$ are out-of-plane polarization parameters [47], $a_{mx}$ describes the influence of the interface spin-mixing conductance on the transmitted in-plane spin current [50], $J_{C,\mathrm{TB}}$ is the electric current density at the interface, n is the interface normal, and $m_{\mathrm{RL}(\mathrm{FL})}$ is the local value of the RL(FL) magnetization at the interface.

#### 3.2.2. Spin Hall Effect

When a charge current flows through an HM layer with strong spin–orbit coupling, it generates a spin current perpendicular to it, carrying a spin polarization perpendicular to the direction of both spin and charge currents [12]. This process is known as the spin Hall effect (SHE). If the FL is deposited right above the HM, this spin current can be employed to provide the torque necessary for switching.

In order to reproduce the SHE, the following term must be added to the spin current expression (9) [12,16]:

(27) $${\tilde{J}}_{S,\mathrm{SHE}}=-\theta_{\mathrm{SHA}}\frac{\mu_{B}}{e}\;\varepsilon \;J_{C}$$

where $\theta_{\mathrm{SHA}}$ is the spin Hall angle, and ε is the rank-3 unit antisymmetric tensor [16]. With the boundary condition

(28) $$\left(\nabla S\right)n=-\theta_{\mathrm{SHA}}\frac{D_{e}}{\sigma }\frac{\mu_{B}}{e}\left(\varepsilon \;J_{C}\right)n$$

the weak formulation with the updated spin current expression is the same as (22) with the addition of the following RHS term:

(29) $${\mathrm{RHS}}_{\mathrm{SHE}}=-\int_{\mathrm{HM}}\theta_{\mathrm{SHA}}\frac{\mu_{B}}{e}(\;\varepsilon \;J_{C}):\nabla vdx$$

The integral is performed only over the HM layer.

#### 3.2.3. Complete Weak Formulation

The complete weak formulation of the spin accumulation equation takes the form

(30) $$\begin{array}{c} D_{e}\int_{\Omega }\left(\nabla S-\beta_{\sigma }\beta_{D}m⊗\left({\left(\nabla S\right)}^{T}m\right)\right):\nabla vdx+\\ +D_{e}\int_{\Omega }\left(\frac{S}{\lambda_{sf}^{2}}+\frac{S\times m}{\lambda_{J}^{2}}+\frac{m\times \left(S\times m\right)}{\lambda_{\varphi }^{2}}\right)\cdot vdx=\\ -\frac{\mu_{B}}{e}\beta_{\sigma }\int_{\omega }\left(m⊗J_{C}\right):\nabla vdx+\frac{\mu_{B}}{e}\beta_{\sigma }\int_{\partial \Omega \cap \partial \omega }\left(\left(m⊗J_{C}\right)n\right)\cdot vdx+\\ -\int_{RL|TB}J_{S,\mathrm{TB}}\cdot v\;dx+\int_{TB|FL}J_{S,\mathrm{TB}}\cdot v-\int_{\mathrm{HM}}\theta_{\mathrm{SHA}}\frac{\mu_{B}}{e}\;(\varepsilon \;J_{C}):\nabla vdx \end{array}$$

where S is the spin accumulation, m is the unit magnetization vector, $J_{C}$ is the charge current density, $J_{S,\mathrm{TB}}$ is the tunneling spin current (26), $D_{e}$ is the electron diffusion coefficient, $\beta_{\sigma }$ and $\beta_{D}$ are polarization parameters, $\lambda_{\mathrm{sf}}$ is the spin-flip length, $\lambda_{J}$ is the exchange length, $\lambda_{\varphi }$ is the dephasing length, and $\theta_{\mathrm{SHA}}$ is the spin Hall angle. The system of linear equations to be solved in the FE implementation of (30) is

(31) $$\underline{A_{S}}\;\underline{S_{h}}=\underline{f_{S}}\;,$$

where $\underline{A_{S}}\in R^{3N}\times R^{3N}$ is the matrix coming from the LHS of (30) and $\underline{f_{S}}\in R^{3N}$ is the vector coming from the RHS. The solution of this system of equations is computed through a solver based on the generalized minimal residual (GMRES) method [51], provided by the library MFEM. The GMRES method is designed for indefinite non-symmetric systems of linear equations, as is the case for (31) due to the presence of the cross-product terms in (30). Material parameters that can change between the different subdomains are treated as piecewise constant coefficients.

As is the case for (14), the inclusion of the additional boundary conditions (25) in the MFEM implementation demands special care. The computation of the boundary terms requires knowledge of the magnetization vector on the opposite interfaces. In order to obtain access to these values, the coefficient describing the boundary integral is initialized as follows:

- For each integration point on the RL|TB interface requiring the computation of the tunneling spin current, the solver loops through the integration points of the TB|FL interface.
- The TB|FL point with coordinates closest to the RL|TB one is selected.
- The integration point number and the element number associated with the found TB|FL point are mapped to the coordinates of the RL|TB one.
- The mapping procedure is repeated for the TB|FL interface.

In a transient simulation, the search is carried out only during the initialization of the solver. At every time step, the data necessary for the computation of (26) can be accessed through the generated maps, without the need to repeat the search procedure.

### 3.3. Magnetization Dynamics Solution

In the tangent plane scheme, the quantity being solved for is the magnetization derivative ∂m/∂t=v, with the constraint m·v=0. By cross-multiplying (1) with m, using the product rule $a\times \left(b\times c\right)=\left(c\cdot a\right)b-\left(a\cdot b\right)c$ and the constraint |m|=1, the LLG equation can be rewritten in a form employed to derive a weak formulation for the tangent plane scheme:

(32) $$\begin{array}{c} \alpha \frac{\partial m}{\partial t}+m\times \frac{\partial m}{\partial t}=\left|\gamma \right|\mu_{0}\;H_{\mathrm{eff}}+\frac{D_{e}}{M_{S}\lambda_{J}^{2}}S+\frac{D_{e}}{M_{S}\lambda_{\varphi }^{2}}m\times S+\\ -\left|\gamma \right|\mu_{0}\left(m\cdot H_{\mathrm{eff}}\right)m-\frac{D_{e}}{M_{S}\lambda_{J}^{2}}\left(m\cdot S\right)m \end{array}$$

The magnetization is taken to belong to $H^{1}$, while the solution v and the test functions w are restricted to a space of vectors orthogonal to the magnetization, $U_{T}=w\in H^{1}\;|\;m\cdot w=0$. The weak formulation of (32) is then

(33) $$\begin{array}{c} \int_{\omega }\left(\alpha v+m\times v\right)\cdot wdx=\left|\gamma \right|\mu_{0}\int_{\omega }\left(H_{\mathrm{ext}}+H_{\mathrm{exc}}+H_{\mathrm{ani}}+H_{\mathrm{demag}}\right)\cdot wdx+\\ +\frac{D_{e}}{M_{S}}\int_{\omega }\left(\frac{S}{\lambda_{J}^{2}}+\frac{m\times S}{\lambda_{\varphi }^{2}}\right)\cdot w\;dx, \end{array}$$

where the last two terms on the RHS of (32) are not present, as their scalar product with the test functions, belonging to the tangent space $U_{T}$, is zero. By using Gauss’s theorem, the weak form of expression (2) for the exchange contribution can be written as

(34) $$\frac{2A_{\mathrm{exc}}}{\mu_{0}M_{S}}\int_{\omega }\nabla^{2}m\cdot wdx=-\frac{2A_{\mathrm{exc}}}{\mu_{0}M_{S}}\int_{\omega }\nabla m:\nabla wdx+\frac{2A_{\mathrm{exc}}}{\mu_{0}M_{S}}\int_{\partial \omega }\left(\left(\nabla m\right)n\right)\cdot wdx$$

The natural Neumann condition (∇m)n=0 is assumed on ∂ω, so that the boundary integral on the RHS is put to zero.

With the given weak formulation, the time derivative v at a certain time $t^{k}$ is obtained by setting [33]

(35) $$m^{k+1}=m^{k}+\theta \delta tv\;,$$

where δt indicates the time step and θ is a parameter ranging from 0 to 1. A value of 0 leads to a fully explicit scheme, while a value of 1 gives a fully implicit one. The value of θ can differ between each effective field contribution. In the implementation reported here, only the exchange field contribution is treated implicitly with θ=1, as this leads to a better stability of the scheme [52]. The weak formulation employed by the FE solver to compute the magnetization dynamics is then expressed as

(36) $$\begin{array}{c} \int_{\omega }\left(\alpha v+m^{k}\times v\right)\cdot wdx+\frac{2A_{\mathrm{exc}}\left|\gamma \right|}{M_{S}}\delta t\int_{\omega }\nabla v:\nabla wdx=\\ -\frac{2A_{\mathrm{exc}}\left|\gamma \right|}{M_{S}}\int_{\omega }\nabla m^{k}:\nabla wdx+\gamma_{0}\int_{\omega }\left(H_{\mathrm{ext}}+H_{\mathrm{ani}}+H_{\mathrm{demag}}\right)\cdot wdx+\\ +\frac{D_{e}}{M_{S}}\int_{\omega }\left(\frac{S^{k}}{\lambda_{J}^{2}}+\frac{m^{k}\times S^{k}}{\lambda_{\varphi }^{2}}\right)\cdot wdx\;, \end{array}$$

(37) $$\begin{array}{c} m^{k+1}=\frac{m^{k}+\delta t\;v}{|m^{k}+\delta t\;v|}\;, \end{array}$$

with the initial condition $m\left(0\right)=m^{0}$. Equation (37) is evaluated nodewise. The additional tangent plane constraint m·w=0 leads to the following saddle point problem [53]:

(38) $$\left(\begin{array}{cc} \underline{A_{M}} & \underline{C_{M}^{T}}\\ \underline{C_{M}} & \underline{0} \end{array}\right)\left(\begin{array}{c} \underline{v_{h}}\\ \underline{\lambda } \end{array}\right)=\left(\begin{array}{c} \underline{f_{M}}\\ \underline{0} \end{array}\right)$$

where $\underline{A_{M}}\in R^{3N}\times R^{3N}$ is the matrix coming from the LHS of (36), $\underline{f_{M}}\in R^{3N}$ is the vector coming from its RHS, $\underline{\lambda }$ is a scalar field, and $\underline{C_{M}}\in R^{N}\times R^{3N}$ implements the constraint. A solution of (38) is computed at each time step through a solver based on the GMRES method.

### 3.4. Demagnetizing Field

The demagnetizing field contribution needs to be computed from the magnetic potential as (4), which in turn is obtained from (5). A direct FE implementation of the latter requires a large computational domain surrounding the magnetic material in order to ensure the proper decay properties of the computed potential. There have been various solutions proposed to solve this open-boundary problem [54,55,56,57], with the truncation of the external domain surrounding the magnetic one at a certain distance being the most straightforward. This approach, however, decreases computational efficiency, as it requires the inclusion of additional degrees of freedom.

High accuracy and reduced computational costs can be achieved by employing a hybrid approach, combining the FE method with the boundary element method (FEM-BEM), allowing $u_{m}$ to be computed only in the magnetic subdomains [58]. The potential is first split into two parts:

(39) $$u_{m}=u_{m,1}+u_{m,2}$$

$u_{m,1}$ satisfies (5) inside the magnetic subdomain, with the boundary condition $\nabla u_{m,1}\cdot n=M_{S}\;m\cdot n$, and is zero outside of it. $u_{m,2}$ satisfies the Laplace equation

(40) $$\nabla^{2}u_{m,2}=0\;,$$

with the boundary conditions $[\nabla u_{m,2}\cdot n]=0$ and $\left[u_{m,2}\right]=u_{m,1}$, where […] denotes a discontinuity across the boundary. By having $u_{m,2}\to 0$ for |x|→∞, potential theory leads to the following relation between $u_{m,1}$ and $u_{m,2}$ [59]:

(41) $$u_{m,2}=\int_{\partial \omega }u_{m,1}\frac{\partial }{\partial n}\frac{1}{|x^{\prime }-x|}dx^{\prime }$$

The decomposition allows $u_{m,1}$ to be computed by solving (5) only in the disconnected magnetic layers. The boundary value of $u_{m,2}$ is obtained by solving (41), and is then used as a Dirichlet condition for (40), which is also computed only inside the magnetized portions of the structure.

The weak formulation of (5), after applying Gauss’s theorem and the boundary condition, results in

(42) $$\int_{\omega }\nabla u_{m,1}\cdot \nabla vdx=M_{S}\int_{\omega }m\cdot \nabla vdx\;,$$

while that of (40) is

(43) $$\int_{\omega }\nabla u_{m,2}\cdot \nabla vdx=0$$

Both the magnetic potential and the test functions belong to $H^{1}$. The FE implementation results in a system of equations analogous to the one employed for the charge potential.

A BEM approach is employed to discretize (41) on the magnetic boundary, resulting in the following matrix-vector multiplication:

(44) $$\underline{u_{m,2}^{\mathrm{bdr}}}=\underline{B_{M}}\;\underline{u_{m,1}^{\mathrm{bdr}}}$$

The matrix $\underline{B_{M}}$ belongs to $R^{N_{\mathrm{bdr}}}\times R^{N_{\mathrm{bdr}}}$ and $\underline{u_{m,1}^{\mathrm{bdr}}}$, $\underline{u_{m,2}^{\mathrm{bdr}}}$ belong to $R^{N_{\mathrm{bdr}}}$, with $N_{\mathrm{bdr}}$ being the number of boundary nodes of the magnetic subdomains. Even though $\underline{B_{M}}$ is a dense matrix, the employment of matrix compression algorithms [60] can significantly reduce the memory demands [58]. The matrix compression algorithms and BEM functionalities are implemented by employing the H2Lib library [61]. The demagnetizing field is finally computed as the gradient of the magnetic potential $u_{m}$ by using the same projection approach employed for the charge current in (20).

**Figure 3:**
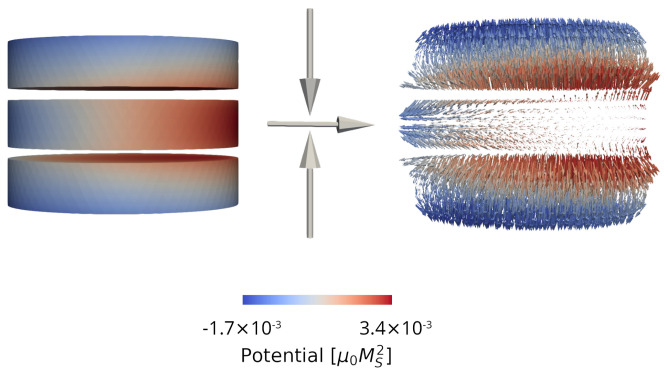
Magnetic potential (**left**) and demagnetizing field (**right**) computed in a structure with three disconnected ferromagnetic layers. The magnetization orientation in each layer is indicated by the arrows. The color coding indicates the value of the magnetic potential.

An example of the magnetic potential and field computed in a structure with three disconnected ferromagnetic layers is shown in Figure 3. Without interaction between the layers, the potential would only vary linearly along the magnetization direction. When applying the described FEM-BEM approach, the interactions are taken into account and the magnetic potential in each layer is shifted due to the stray field contributions of the neighboring ferromagnetic segments. The presented implementation allows the demagnetizing field acting in structures containing multiple ferromagnetic layers, as is typical of modern MRAM cells, to be readily computed.

## 4. Device Simulation

Recently proposed devices are composed of several layers of ferromagnetic materials, non-magnetic spacers, and tunnel barriers, in order to reduce switching currents and cell size. Due to the capability of computing the torque acting in all layers from a unified expression, the presented FE solver is suitable for the simulation of such structures. The following sections report the results of switching simulations performed in the structures of Figure 1. The parameters employed are presented in Table 1. They are consistent with CoFeB and MgO for the FM layers and TB layers, respectively. The low values of $\lambda_{J}$ and $\lambda_{\varphi }$ are employed to have complete absorption of the transverse spin accumulation components near the TB interface [46]. The results reported in this paper were obtained by employing tetrahedral elements.

### 4.1. Double RL STT-MRAM

In order to reduce the critical current required for switching, an additional RL (RL${}_{2}$) can be deposited on top of the FL [20] (cf. Figure 1b). When RL${}_{2}$ is anti-parallel to the first RL (RL${}_{1}$), the torque coming from the two becomes additive, and the switching is made faster [62]. To not compromise the TMR and data read, the second RL is separated from the FL by a non-magnetic metallic spacer (NMS).

**Figure 4:**
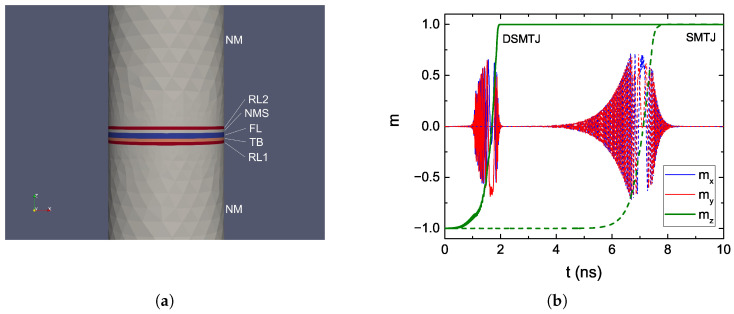
(**a**) Structure for an MRAM cell with the addition of a second RL (RL2), separated from the FL by a non-magnetic metallic spacer (NMS). The RL${}_{1}$, RL${}_{2}$, and TB are 1 nm thick, the FL is 1.7 nm thick, and the NM contacts are 50 nm thick. (**b**) Magnetization reversal of the FL from AP to P for an MRAM cell with a single MTJ (SMTJ, dotted line) and the one with a double RL (DSMTJ, solid line).

We employed the presented solver to perform an AP to P switching simulation of both a regular MTJ with single RL (SMTJ) and the double RL MTJ (DSMTJ). The structure used for the DSMTJ simulation, with a diameter of 40 nm, is reported in Figure 4a. Long NM contacts were employed to allow the spin accumulation to completely decay inside them. The total number of nodes in the mesh was 7338. The magnetization reversal of the FL in both the SMTJ and DSMTJ is reported in Figure 4. A voltage of 1.0 V was applied. We note that the switching of the DSMTJ, presenting more oscillations in the z-component, is less smooth than the one of the SMTJ. This is due to the additional torque and stray field contributions from the second RL, which cause the magnetization to switch less uniformly and to produce the observed non-smooth trajectory of its average components. The results show a substantial reduction in switching time for the same applied voltage in the DSMTJ, in good agreement with the experimental results reported in [20].

### 4.2. Ultra-Scaled STT-MRAM

The stability of the FL can be increased by adding additional MgO tunneling layers, because of the perpendicular anisotropy provided by their interfaces with CoFeB. Moreover, employing elongated layers with small diameters allows additional stability to be gained from the contribution of the shape anisotropy [21] (cf. Figure 1c). Because of the reduced FL diameter, the scalability of this kind of device is also improved.

**Figure 5:**
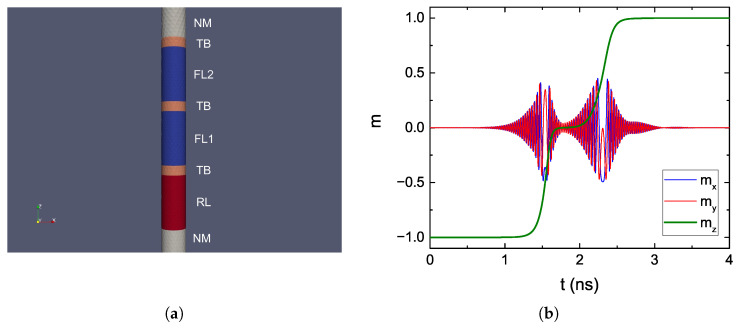
(**a**) Structure for an elongated MRAM cell with FL composed of two sections (FL1 and FL2), separated by a TB. The RL, FL${}_{1}$, and FL${}_{2}$ are 5 nm thick, all the TBs are 0.9 nm thick, and the NM contacts are 50 thick. (**b**) Magnetization reversal of the FL from AP to P for the elongated cell.

We employed the FE solver to investigate the switching behavior of such ultra-scaled MRAM cells. The structure used for the simulation is reported in Figure 5a. The cell had a diameter of 2.3 nm, and the total number of nodes was 9634. The FL was capped by a second TB, and further split into two sections, FL${}_{1}$ and FL${}_{2}$, by a third TB. The applied bias voltage was 1.5 V. The magnetization reversal from AP to P computed in this mesh is reported in Figure 5b, where clear steps in the trajectory can be observed. This is caused by the fact that while the static magnetic coupling between the two segments increases the overall stability of the FL and allows them to respond coherently to an applied external field, during STT switching the torque contributions coming from the different TBs make the segments switch one at a time. At the beginning of the process, the torques acting from RL and FL${}_{2}$ on FL${}_{1}$ are additive, causing it to switch first and fast. Then, the torque acting from FL${}_{1}$ on FL${}_{2}$ makes it switch as well, at a slower pace. The observed behavior can help explain the reduction in critical switching current observed for a quad-interface device in [63].

### 4.3. SOT Assisted STT-MRAM

Including the SHE in the model allows for the proper treatment of SOT. By interfacing the FL with an HM layer, and running a second current through it, it is possible to assist the STT switching by bringing the magnetization in-plane with SOTs in the starting phase [64] (cf. Figure 1d). With SOT, the incubation time needed for the FL to break its colinearity with the RL is avoided, reducing the overall switching time at the cost of a larger footprint.

**Figure 6:**
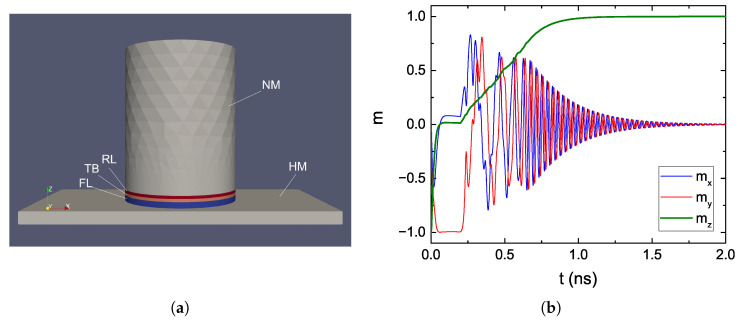
(**a**) Structure reproducing an SOT + STT-based MRAM cell. The MTJ stack is deposited on top of a heavy metal line (HM). The RL and TB are 1 nm thick, the FL is 2 nm thick, the top NM contact is 50 nm thick, and the HM layer is 4 nm thick, 50 nm wide and 100 nm long. (**b**) Magnetization reversal of the FL from AP to P for the SOT + STT cell.

We applied the presented solver to the switching simulation of an MRAM cell relying on both STT and SOT. The structure used for the simulation is reported in Figure 6a. The diameter of the MTJ is 40 nm, and the total number of nodes is 15,058. A bias voltage of 2.0 V was used for the STT current and one of 0.3 V for the SOT current. The parameters employed for the HM are consistent with Pt, with a spin Hall angle of 0.19 [65]. The SOT current is only applied for the first 0.2 ns of the simulation. As expected, the magnetization is quickly brought in-plane by the SOT contribution. When the SOT current is turned off, the switching is completed by the STT contribution.

## 5. Conclusions

We presented the derivation of a finite element solution to the weak formulation of the LLG equation coupled with the spin and charge drift-diffusion formalism. The treatment of the tunneling layers as poor conductors, whose local conductivity depends on the relative magnetization orientation in the ferromagnetic layers, and the addition of appropriate boundary conditions at the tunnel barriers’ interfaces, can account for the properties of both resistance and torque expected in MTJs. The addition of terms accounting for the SHE to the spin equation allows us to also reproduce the contribution of spin-orbit torques. The demagnetizing field is computed by employing a hybrid FEM-BEM approach. The presented solver was successfully used to perform switching simulations of recently proposed structures composed of several ferromagnetic, tunneling and non-magnetic layers, as well as heavy metal lines for the generation of spin-orbit torques, supporting its employment to help investigate and predict the switching performance of newly introduced devices.

## Figures and Tables

**Table 1:** Material parameters.

LLG parameters	Value
Saturation magnetization ($M_{s}$)	$0.81\times 10^{6}$ A/m
Exchange constant ($A_{exc}$)	$2.0\times 10^{-11}$ J/m
Interface anisotropy ($K_{int}$)	$1.29\times 10^{-3}$ J/m${}^{2}$
Gilbert damping constant (α)	0.02
Drift-diffusion parameters	Value
Conductivity polarization, $\beta_{\sigma }$	0.52
Diffusion polarization, $\beta_{D}$	0.7
FM diffusion coefficient, $D_{e,FM}$	$10^{-3}$ m${}^{2}$/s
NM diffusion coefficient, $D_{e,NM}$	$10^{-2}$ m${}^{2}$/s
HM diffusion coefficient, $D_{e,HM}$	$1.1\times 10^{-3}$ m${}^{2}$/s
TB diffusion coefficient, $D_{S}$	$2.0\times 10^{-8}$ m${}^{2}$/s
FM conductivity $\sigma_{FM}$	$4.0\times 10^{6}$ S/m
NM conductivity $\sigma_{NM}$	$5.0\times 10^{6}$ S/m
HM conductivity $\sigma_{HM}$	$7.0\times 10^{6}$ S/m
FM spin-flip length, $\lambda_{sf,FM}$	10 nm
NM spin-flip length, $\lambda_{sf,NM}$	10 nm
HM spin-flip length, $\lambda_{sf,HM}$	1.4 nm
Spin exchange length, $\lambda_{J}$	0.8 nm
Spin dephasing length, $\lambda_{\varphi }$	0.4 nm
Spin Hall angle, $\theta_{\mathrm{SHA}}$	0.19
TB resistance standard and double RL STT-MTJ	Value
Resistance parallel ($R_{P}$)	$4.3\times 10^{3}$ kΩ
Resistance anti-parallel ($R_{AP}$)	$9.1\times 10^{3}$ kΩ
TB resistance ultra-scaled STT-MTJ	Value
Resistance parallel ($R_{P}$)	$4.1\times 10^{5}$ kΩ
Resistance anti-parallel ($R_{AP}$)	$7.5\times 10^{5}$ kΩ
TB resistance SOT-assisted STT-MTJ	Value
Resistance parallel ($R_{P}$)	$1.4\times 10^{4}$ kΩ
Resistance anti-parallel ($R_{AP}$)	$4.2\times 10^{4}$ kΩ

## Data Availability

The datasets generated during and/or analyzed during the current study are available from the corresponding author on reasonable request.

## Abbreviations

The following abbreviations are used in this manuscript:

MRAM	Magnetoresistive random access memory
CMOS	Complementary metal-oxide semiconductor
SRAM	Static random access memory
MTJ	Magnetic tunnel junction
FM	Ferromagnetic
NM	Non-magnetic
RL	Reference layer
FL	Free layer
TB	Tunnel barrier
NMS	Non-magnetic spacer
HM	Heavy metal
P	Parallel
AP	Anti-parallel
TMR	Tunneling magnetoresistance ratio
STT	Spin-transfer torque
SOT	Spin-orbit torque
SHE	Spin Hall effect
LLG	Landau–Lifshitz–Gilbert
FE	Finite element
CRMT	Continuous random matrix theory
BEM	Boundary element method
LHS	Left-hand side
RHS	Right-hand side
DSMTJ	Double spin torque MTJ
